# Sequence Information Encoded in DNA that May Influence Long-Range Chromatin Structure Correlates with Human Chromosome Functions

**DOI:** 10.1371/journal.pone.0002643

**Published:** 2008-07-09

**Authors:** Taichi E. Takasuka, Alfred Cioffi, Arnold Stein

**Affiliations:** Department of Biological Sciences, Purdue University, West Lafayette, Indiana, United States of America; Pasteur Institute, France

## Abstract

Little is known about the possible function of the bulk of the human genome. We have recently shown that long-range regular oscillation in the motif non-T, A/T, G (VWG) existing at ten-nucleotide multiples influences large-scale nucleosome array formation. In this work, we have determined the locations of all 100 kb regions that are predicted to form distinctive chromatin structures throughout each human chromosome (except Y). Using these data, we found that a significantly greater fraction of 300 kb sequences lacked annotated transcripts in genomic DNA regions ≥300 kb that contained nearly continuous chromatin organizing signals than in control regions. We also found a relationship between the meiotic recombination frequency and the presence of strong VWG chromatin organizing signals. Large (≥300 kb) genomic DNA regions having low average recombination frequency are enriched in chromatin organizing signals. As additional controls, we show using chromosome 1 that the VWG motif signals are not enriched in randomly selected DNA regions having the mean size of the recombination coldspots, and that non-VWG motif sets do not generate signals that are enriched in recombination coldspots. We also show that tandemly repeated alpha satellite DNA contains strong VWG signals for the formation of distinctive nucleosome arrays, consistent with the low recombination activity of centromeres. Our correlations cannot be explained simply by variations in the GC content. Our findings suggest that a specific set of periodic DNA motifs encoded in genomic DNA, which provide signals for chromatin organization, influence human chromosome function.

## Introduction

Very little is known about the possible function of the bulk of the human genome, and we must be open to the investigation of novel, perhaps unexpected, ways in which DNA sequence can confer biological function [1]. Because essentially all of the nuclear DNA is packaged into chromatin, it is likely that functional effects of non-coding DNA are mediated by the chromatin structure. Recently, a “code” for nucleosome positioning was reported for yeast, and evidence was provided that it is involved in gene regulation [2], [3]. It has been further suggested that sequence information of various types influences many cellular processes, and a current goal is to find how sequence information encoded in genomic DNA influences large-scale features of DNA packaging [4].

Recent advances in chromatin structure provide new insights into how higher-order chromatin structure might confer biological function. Previously, it was presumed that essentially all of the transcriptionally inactive nuclear DNA was packaged into a uniform solenoid-like structure [5], [6]. However, the existence of a single regular chromatin structure was questioned [7], [8], and recently the solenoid model was essentially disproved [9], [10]. For non-solenoid models, the particular higher-order chromatin structure that is formed necessarily depends upon the arrangement of the nucleosomes within a nucleosome array. Nucleosome linker lengths, the DNA lengths between adjacent nucleosomes, vary [11], and therefore non-solenoid models can generate a large number of different chromatin higher-order structures [12], consistent with the visualization of a variety of intricately folded structures containing bends, twists and loops by cryo-EM [12] and scanning force microscopy [13].

It is likely that the nucleosome arrangement in an array is not formed randomly. Instead, periodicities in the DNA sequence could very well lead to the formation of a particular nucleosome arrangement, which could then form a distinctive higher-order structure or, at least, have a distinctive mode of flexibility [12], [14]. These structural/physico-chemical differences could be functionally important. For example, with this mechanism, it would be easier to understand how histone modifications and the presence of non-histone chromosomal proteins can remodel chromatin [15]. Chromatin remodeling might then involve making only subtle alterations to a distinctive chromatin higher-order structure already possessing transcription factor binding sites in close proximity in three dimensional space.

Recently, our laboratory showed that the triplet motif non-T, A/T, G (VWG) [16] or its complement (CWB) contributes to a genomic DNA code for chromatin structure, at least in higher organisms where histone H1 is present [17]. There are ten VWG/CWB trinucleotides: AAG, GAG, CAG, ATG, GTG, CTG, CAT, CAC, CTT, and CTC. Period-10 (10.00–10.33) occurrences in the motifs were counted in a sliding window that extended ± 51 nucleotides (5 periods) from each VWG/CWB position. The counts were averaged in a 60 nucleotide window that slid 5 nucleotides per step to generate a continuous curve of VWG/CWB counts as a function of nucleotide number. The strengths of regular dinucleosome-length (333 bp to 400 bp) oscillations in period-10 VWG/CWB occurrences were assessed over 100 kb regions by Fourier analysis. A large Gaussian-like Fourier amplitude at a dinucleosome period was considered to be a prospective signal for forming a distinctive nucleosome array. We used the mouse genomic DNA sequence to test our ability to computationally predict 100 kb regions of the mouse genome that exhibit unusually short (<185 bp) nucleosome array periodicities, compared with the bulk chromatin periodicity of 195±5 bp, in mouse liver nuclei. We tested each prediction experimentally and showed that we were able to correctly predict 10 out of 10 nucleosome array periodicities ±5 bp (*P*-value<10^−7^) [17]. Our findings suggested that it should now be possible, for the first time, to predict which regions of the human genome have more limited or defined, rather than random or highly variable, chromatin structures.

In this work, we have determined the locations of all 100 kb regions that have strong VWG nucleosome array formation signals, and therefore may form distinctive chromatin structures, throughout the human genome. We then examined the Ensembl transcript annotations in 300 kb sequences from the centers of all genomic DNA regions of size ≥300 kb that were signal-rich for comparison with 300 kb regions from the centers of ≥1 Mb regions that lacked signals. We found a statistically significant difference in the annotated transcript contents between the two datasets, consistent with the idea that large non-transcribed regions have distinctive chromatin structures. We next made use of the recently provided genome-wide very high resolution meiotic recombination frequency data [18] to see if there is evidence for a possible relation between meiotic recombination frequency and DNA sequence-encoded chromatin structure. We found that genomic DNA regions of low meiotic recombination frequency are enriched in VWG chromatin signals. Most currently available databases useful for assessing human chromosome functions other than transcription and meiotic recombination frequency were not of high enough resolution for this analysis. We also provide evidence that centromeric DNA, which has very low recombination rates [19], [20], has particularly strong VWG signals that are consistent with the unusual nucleosome repeat that has been observed experimentally in a portion of centromeric chromatin. Our findings cannot be explained simply by GC-content variations in the genome [21]–[23], and suggest that long-range periodic VWG signals influence chromatin structure and chromosome function.

## Results

### Genomic DNA from Signal-rich Regions Contain a Higher Percentage of Central Three Hundred kb Sequences that Lack Transcripts than those from Signal-poor Regions

**Figure 1:**
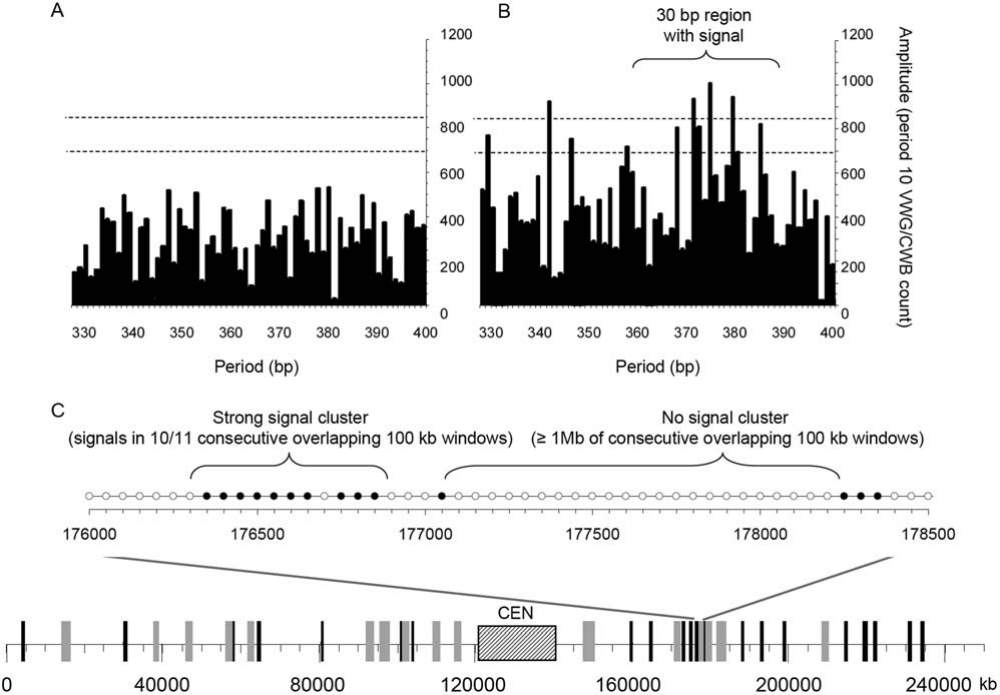
Analysis of Genomic DNA for VWG/CWB Chromatin Organizing Signals. Examples of Fourier transforms of period-10 VWG/CWB oscillations in 100 kb windows for no signal (A) and a strong signal (B). The Fourier amplitude value is computed at each base pair in the physiological dinucleosome range. For a signal we require that at least 5 Amplitude values within a 30 bp region are ≥700 (lower dashed horizontal line). In addition, we require that at least one of the central 3 periods has an amplitude value ≥850 (upper horizontal dashed line), thereby approximating a Gaussian distribution of amplitude values over the ≤30 bp region. (C) The distribution of (≥300 kb) signal-rich or (≥1 Mb) signal-poor clusters across chromosome 1, as described in the text (lower diagram). The black bars and grey bars indicate the positions and relative sizes of the signal clusters and the regions lacking signals, respectively. The patterned rectangle indicates the position of the centromere. The scale below the diagram represents the coordinates along the chromosome in kb. An expanded 2.5 Mb region from is shown above the chromosome 1 diagram. In the expanded region, filled circles and open circles denote the centers of the 100 kb windows containing or lacking a signal, respectively. The region shown contains one 600 kb region with nearly consecutive signals in each 100 kb window sliding in 50 kb steps and one >1 Mb region that lacked a signal in each consecutive 100 kb window sliding in 50 kb steps. Other parts of the 2.5 Mb-region do not contain nearly continuous signals for ≥300 kb or regions ≥1 Mb that lack signals.

To identify regions predicted to contain sequence-defined long-range chromatin structures, Fourier transforms of the period-10 VWG/CWB oscillations in 100 kb windows were computed across all of the human chromosomes except for Y, as described in Materials and Methods. The 100 kb windows slid in 50 kb steps, and are regarded as being statistically independent of each other. These results are available in the Table S1. In Fig. 1A and 1B, an example of a signal-lacking 100 kb region and a signal-containing 100 kb region, respectively, is shown. The signal strength (Fourier Amplitude value) in the physiological dinucleosome period range (330 bp–400 bp) is assessed. Based upon our previous experimental work [17], we defined a signal as one that has an approximately Gaussian Amplitude peak distribution with a standard deviation of about 7 to 15 bp and a minimum amplitude of at least 700 period-10 VWG/CWB counts (lower horizontal dashed line on the Amplitude axes). Here, the Fourier Amplitude value is computed at each base pair value, and to meet approximately the same criterion for a signal that we previously determined, we require for a signal that at least 5 Amplitude values within a 30 bp region are ≥700, and that at least one of the central 3 periods has an Amplitude value ≥850 (upper horizontal dashed line on the Amplitude axes), thereby approximating a Gaussian distribution. For chromosomes 1–22 plus X, there were 10,596 signals found in 51,833 (100 kb) windows (giving a “genome average” value of 0.204 signals per window).

Larger genomic DNA regions (≥300 kb) that contained clusters of nearly-consecutive signals, as well as regions ≥1 Mb that completely lacked signals were also identified. These results are available in Table S2. Signal-rich clusters of size greater than 5 consecutive overlapping windows (300 kb) were allowed to contain one gap. For example: 5/6, 6/7, 7/8, etc. signals per group of overlapping 100 kb windows or better were allowed. In Fig. 1C, the occurrence, distribution, and relative sizes of the ≥300 kb clusters of nearly-consecutive signals (black bars) and ≥1 Mb clusters with no signals (grey bars) across chromosome 1 are shown as an example. The higher resolution shown in the expanded 2.5 Mb region indicates the presence of one ≥1 Mb no-signal cluster and the presence of a 600 kb signal cluster with 10/11 overlapping 100 kb windows (with a 50 kb slide) possessing a signal. For chromosomes 1–22 combined, there were 193 signal-rich and 275 signal-poor regions, as defined here. Because of the relatively low fraction (0.20) of windows containing signals, we chose a larger cluster size for the signal-poor regions than for the signal-rich regions in order to make the numbers of entries in the two datasets nearly equal. Additionally, using 300 kb signal-rich and signal-poor regions should more reliably reflect large-scale chromatin structures than by using individual 100 kb windows.

We next examined Ensembl annotations in the 300 kb regions taken from the center of each signal-rich and signal-poor region. It is plausible that ≥300 kb regions that are not transcribed in any cell type may have different chromatin structures from those ≥300 kb regions that do produce transcripts. This size should be large enough to form relatively stable higher-order chromatin structures, if such structures exist. We flagged only those 300 kb regions that were annotated to have no gene or pseudogene transcripts of any kind, no non-coding RNA transcripts, no micro RNA (or other small RNA) transcripts, and no ESTs. The chromosome-by-chromosome results are available in Table S2.

Of the 468 (the total number of) 300 kb regions analyzed, only 38 (8.1%) were annotated to not contain transcripts. This low percentage is because transcripts, as defined here, are abundant in the genome, and 300 kb is a rather large region. Of these 38 non-transcribed regions, 24 out of the 193 analyzed (12%) were in the signal-rich dataset, whereas only 14 out of the 275 analyzed (5.1%) were in the signal-poor data set, corresponding to a greater than 2-fold increase in the signal-rich over the signal-poor dataset. Although the numbers of 300 kb sequences annotated to include no transcripts of any kind are necessarily small, the higher percentage in the signal-rich dataset compared to the signal-poor dataset is statistically significant (*P*-value = 0.02). This finding is consistent with the idea that non-transcribed sequences may possess distinctive chromatin structures.

### Large Genomic DNA Regions with Low Average Recombination Frequencies are Enriched in VWG/CWB Chromatin Organizing Signals

**Figure 2:**
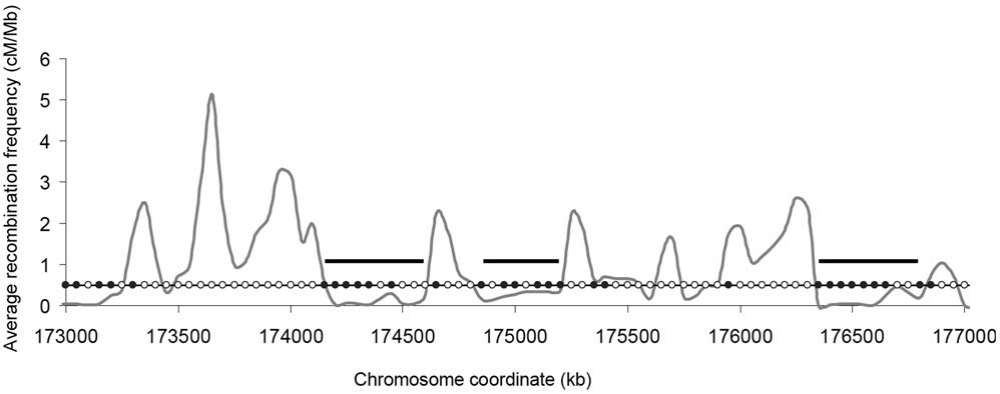
Average Recombination Frequency and Signal Distribution in a Region of Chromosome 1. The grey curve shows the recombination frequency averaged in 100 kb windows that slid in 50 kb steps. The filled circles and open circles denote the centers of overlapping 100 kb windows having a signal or lacking a signal, respectively. The bars indicate the three ≥300 kb regions with average recombination frequencies <0.5 cM/Mb.

Recently, high resolution data on the meiotic recombination frequency throughout the human genome was provided [18]. Approximately 1.6 million single nucleotide polymorphisms (SNPs) were used to deduce the recombination frequency (cM/Mb) every approximately 1000 bp on the average. To determine whether there was a possible relationship between recombination frequency and regions of the genome predicted to potentially form distinctive chromatin structures, we first averaged this high resolution data in a 100 kb window that slid in 50 kb steps across each chromosome. This was the same window size and slide that we used to compute the VWG signals, thereby allowing a comparison between regions of the genome that had high or low recombination frequencies with regions that possessed or lacked chromatin organizing signals. Fig. 2 shows a 4 Mb region of chromosome 1 as an example, which is only a small portion of the total data. It shows that despite the averaging in 100 kb windows, there is still considerable variation from the genome average recombination frequency value of 1.5 cM/Mb. For example there are 2 peaks with average recombination frequency greater than 3 cM/Mb and there are 3 regions of size ≥300 kb where the average recombination frequency is less than 0.5 cM/Mb (black horizontal bars), the value that we defined as the upper cut-off value for low recombination frequencies for this analysis. The circles on the horizontal line at 0.5 cM/Mb in Fig. 2 represent the centers of our overlapping 100 kb windows which either possessed a VWG signal (black circles) or did not (white circles). Strikingly, the DNA regions with higher recombination frequencies seem to avoid the VWG signals, and the regions with low average recombination frequencies, as in the four regions indicated by the horizontal black bars, seem to be enriched in the VWG signals. Although there were many exceptions to this rule genome-wide, it appeared that it might be the general trend by simple inspection of the data.

To determine whether there is a statistically significant enrichment of genomic DNA regions ≥300 kb that have average recombination frequencies ≤0.5 cM/Mb, we first identified all such regions, across each chromosome (except Y), as described in Materials and Methods. We will refer to these regions as recombination cold spots (RCSs). We then calculated the fraction of the 100 kb windows in each RCS that contained a VWG signal for comparison with the chromosome 1–22 total sequence value of 0.208 signals per window. Chromosome X appears to be anomalous (see Discussion), and was not included in this overall average value. As a refinement of this analysis, we corrected for the local variations that occur in the average fraction of 100 kb windows containing VWG signals in the vicinity of each RCS. Although the autosome average value was 0.208 VWG signals per window, we observed values as low as 0.05 and as high as 0.42 when we examined all non-overlapping 1-Mb regions. The autosome standard deviation value was 0.07. No consistent pattern to the variations was found among chromosomes. To correct for the variations that occur in the vicinity of an RCS, we computed the local value of the fraction of 100 kb windows that contained a VWG signal in the 1-Mb regions flanking each RCS. The 100 kb windows slid in 50 kb steps in all computations. The value of the signal fraction within an RCS was then compared to that of the average value of its two 1-Mb flanks. Thus, when the flanking signal fraction was above the autosome average value, the RCS signal fraction was proportionally decreased to account for the locally high value. Conversely, when the flanking signal fraction value was below the autosome average value, the RCS signal fraction was proportionally increased to account for the locally low value. When RCSs were less than 1 Mb apart, the same correction was applied to the cluster of RCSs, based upon the 1-Mb values flanking the cluster.

A summary of the chromosome-by-chromosome analysis is given in Table 1. The details of the analysis are provided in Table S3. It shows that most of the chromosomes had signal fraction values in RCSs that were significantly greater than the genome average of 0.21, as evidenced by the *P*-values. For example, eleven chromosomes had RCS signal fraction values ≥0.30. The exceptions, chromosomes 5, 18, 21, and X will be discussed below (see Discussion). The combined chromosome 1–22 RCS signal fraction value was 0.295 for more than 6,000 windows. The very low *P*-value of <10^−60^ reflects the large number of windows involved.

**Table 1:** Analysis of the human genome and recombination cold spots (RCSs), defined as genomic DNA regions of size ≥300 kb having average recombination frequency ≤0.5 cM/Mb, for VWG chromatin signals in 100 kb windows.

Source	Number of windows analyzed	Number of signals found	Signals/window	*P*-value
Autosomes	49097	10198	0.208	
Ch X	2736	398	0.145	
Ch 1 RCSs	539	160	0.297	<10^−6^
Ch 2 RCSs	773	234	0.303	<10^−9^
Ch 3 RCSs	614	166	0.270	<10^−3^
Ch 4 RCSs	477	136	0.285	<10^−4^
Ch 5 RCSs	431	86	0.200	0.68
Ch 6 RCSs	474	129	0.272	<10^−3^
Ch 7 RCSs	398	119	0.299	<10^−4^
Ch 8 RCSs	343	101	0.294	<10^−4^
Ch 9 RCSs	181	57	0.315	<10^−3^
Ch 10 RCSs	280	113	0.404	<10^−12^
Ch 11 RCSs	362	135	0.373	<10^−12^
Ch 12 RCSs	320	81	0.253	0.03
Ch 13 RCSs	193	81	0.420	<10^−10^
Ch 14 RCSs	186	51	0.274	0.02
Ch 15 RCSs	194	51	0.263	0.04
Ch 16 RCSs	145	40	0.276	0.03
Ch 17 RCSs	172	56	0.326	<10^−3^
Ch 18 RCSs	108	27	0.250	0.17
Ch 19 RCSs	72	23	0.319	0.02
Ch 20 RCSs	108	33	0.306	0.01
Ch 21 RCSs	15	1	0.067	0.97
Ch 22 RCSs	76	24	0.316	0.02
Ch X RCSs	724	74	0.123	0.92
Ch 1-22 RCSs	6461	1904	0.295	<10^−60^

Chromosome Y was excluded from the analysis. The number of signals found was adjusted to account for the influence of the signal densities of the 1-Mb regions flanking each RCS, as described in the text. The *P*-value (obtained from the cumulative binomial distribution) is the probability of obtaining the number of signals found or a greater value by chance.

We also performed this analysis using lower RCS upper cut-off values of 0.2 cM/Mb and 0.1 cM/Mb. The *P*-values necessarily increased as the number of RCSs decreased, but the number of RCS signals per window remained constant at 0.30. The statistical significance remained very high (*P*-value<10^−16^). For chromosome 1, we also investigated the effect of using higher RCS upper cut-off values of 0.70 cM/Mb and 1.0 cM/Mb. As expected, the number of RCS signals per window decreased toward the genome average value of 0.21 at the higher cut-off value. At 0.70 cM/Mb, it was 0.30 (*P*-value<10^−10^), while at 1.0 cM/Mb, it was 0.22 (*P*-value = 0.03).

As a control, we performed the analysis for chromosome 1 using 68 randomly selected DNA regions instead of the 68 RCSs. The size of each randomly chosen region was 450 kb, the mean RCS size. In contrast with the RCS dataset, we found that the number of signals per window from this randomly selected dataset was slightly lower than the genome average value of 0.21. Thus, genomic DNA regions of size ≥300 kb having average recombination frequencies less than about 0.5 to 0.7 cM/Mb are enriched in VWG chromatin signals by more than 40% compared to the autosome average value or to randomly chosen regions having the mean RCS size.

As an additional control, we performed the analysis for chromosome 1 using other motif/complement sets (containing 10 to 12 motifs each, as for VWG/CWB). There are C(32,6) = 906,192 possible different ways of selecting 6 triplet/complement motifs out of the 32 that exist, giving 906,192 different sets containing 10 to 12 unique triplets each. We chose these motif sets indiscriminately; simply requiring that they did not contain many triplet motifs that were also present in the VWG set, or have no motifs in common with the VWG set. Such motif sets would be expected to give periodic oscillations in their occurrence for the same DNA sequences as for VWG just because these motifs either followed or did not follow VWG. For example, in the former case, when the VWG count was high the similar motif set would also be high, while in the latter case, when the VWG count was high, the non-similar motif set count would tend to be low. Table 2 reports: the average number of the period-10 motif indicated per 102 bp, the number of dinucleosome period signals per 100 kb window (defined in the same way as for VWG), the *P*-value assessing the degree of concordance of the motif signal with the VWG signal (lower values indicate more concordance; values close to 0.5 indicate no relation between where the two different signals occur on the chromosome), the number of signals per 100 kb window in RCSs, and the *P*-value for the significance of the difference between the number of signals in RCSs and on the whole chromosome (*P*-values<0.050 are considered as significant).

**Table 2:** Analysis of VWG and other tri-nucleotide motif sets for chromosome 1.

Motif set	Average no.of period-10 motifs/102 bp	Signals/100 kb window on the chrom.^a^	*P*-value^b^ for concordance with VWG	Signals/100 kb window in RCSs^c^	*P*-value^d^
VWG	3.00	0.20	-	0.30	<10^−6^
HST	2.60	0.075	0.090	0.073	0.56
HCW	3.70	0.078	<10^−3^	0.079	0.46
HWC	2.90	0.083	0.26	0.073	0.79
GWV	2.40	0.10	<10^−3^	0.11	0.41
STH	2.70	0.11	0.56	0.09	0.94
VGW	2.30	0.13	<10^−2^	0.15	0.077
MTV	3.30	0.56	<10^−2^	0.55	0.82
HTM	3.40	0.86	0.96	0.85	0.86
VRG	3.20	0.97	0.21	0.96	0.92
VGR	2.60	0.97	0.37	0.97	0.56
TWH	3.60	0.99	0.63	0.99	0.87
AWH	3.60	0.99	0.63	0.99	0.87

V = A/C/G, W = A/T, S = C/G, H = A/T/C, M = A/C, R = A/G; Each motif set included the complements, giving 10 to 12 motifs per set. VWG and non-VWG motifs are listed. ^a^Average fraction of 100 kb windows that contain signals throughout the chromosome. ^b^ *P*-value for the degree of concordance between VWG signals and other motif signals throughout the chromosome. ^c^Average fraction of 100 kb windows that contain signals in RCS (<0.5 cM/Mb). ^d^ *P*-value for the significance of the difference between the signals/window in RCSs and on the whole chromosome.

Several things are apparent from the Table 2 data. First, in disagreement with the original study [16], the frequency of occurrence of the period-10 VWG motif set is not unusually high compared to other sets of 10 to 12 tri-nucleotides. Second, there is no apparent relation between the frequency of the motif occurrence and the fraction of 100 kb windows that possess signals. Third, none of the12 non-VWG motif sets selected for analysis were enriched in RCSs (see Discussion).

### Centromeric DNA Has a Very Low Recombination Frequency and a Very Strong VWG Chromatin Signal

Human chromosome centromeres are embedded in several thousand kilobase pairs of repetitive alpha satellite DNA consisting of diverged tandemly repeated 171 bp monomer units [24]. Meiotic recombination is significantly suppressed in centromeres [19], [25]. Therefore, it was of interest to see whether centromeric DNA contains VWG signals, which might influence its chromatin structure. The presence of VWG chromatin signals in centromere DNA would be consistent with the enrichment of VWG signals that we have found in other genomic DNA regions having low meiotic recombination activity (RCSs). Because of its highly repetitive nature, centromeric DNA has been refractory to sequencing. However, a 579,924 bp contig (NT_024862) from human chromosome 17 is available in the current genome assembly that contains about 17000 bp of higher-order alpha satellite centromeric DNA at the end. To simulate an approximately 100 kb region of centromeric DNA, we strung together six copies of the 16,545 bp centromeric DNA sequence (coordinates 563335–578794) from the end of this contig.

**Figure 3:**
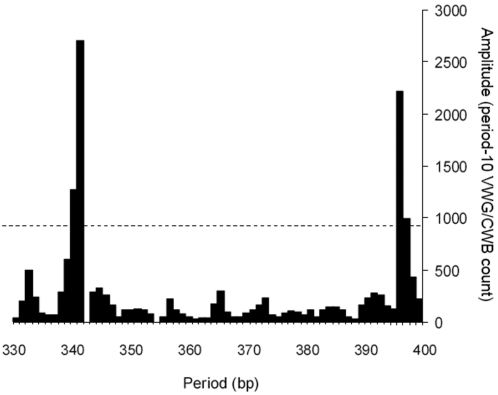
Analysis of Alpha Satellite DNA for VWG/CWB Chromatin Organizing Signals. The Fourier transform of the period-10 VWG/CWB oscillations in an approximately 100 kb window is represented in histogram form, as in Fig. 1. Amplitude values are plotted for every bp throughout the physiological dinucleosome range. The horizontal dashed line indicates the Amplitude value of 850.

The Fourier transform of the VWG/CWB oscillations for this simulated 100 kb centromeric DNA region is shown in Fig. 3. Interestingly, there is a large amplitude (compare with Fig. 1B) at 340 bp, a value close to twice the satellite repeat of 171 bp. Even though there are only two period values within 30 bp that have amplitude values that exceed 850 VWG/CWB counts (dotted line), rather than five period values with amplitudes greater than 700 counts, we think that the large spike at 340 bp constitutes a signal. A narrower distribution of large amplitudes is what should be expected from a signal in repetitive DNA. In addition to the signal at 340 bp, there is another strong dinucleosome signal at 396 bp, predicting a possibly alternative 198 bp nucleosome repeat, a value close to that observed in the bulk chromatin from many cell types [26].

### Effect of the GC-content on the VWG Signal Density and the Correlations

We examined the effect of the GC-content on the VWG signal density, and also addressed the question of whether the correlations that we observed between high signal density and non-transcribed regions, or between high signal density and low recombination frequency could be explained merely by the variables involved having similar GC content variations. For example, signals might be more abundant in regions having higher %GC, and high %GC regions might also have lower recombination frequencies.

The ten VWG/CWB trinucleotides are necessarily more GC-rich (53%) than the human genome average value of 41% [27]. The probability of encountering a VWG/CWB triplet is dependent upon the GC-content of the genomic DNA region under consideration. For example, if the genomic DNA regions under consideration were 0% GC or 100% GC, the probabilities of encountering a VWG/CWB or a signal in either would be zero. The human genome consists of approximately 3200 GC isochores, large DNA regions (>200 kb) that are characterized by internal variations in GC % that are well below the full extent of the genomic DNA variation [28]. There are five GC isochore families: L1, L2, H1, H2, and H3; containing %GC values of <37%, 37%–41%, 41%–46%, 46%–53%, and >53%, respectively. The fraction of the genome contained in each GC isochore family is: 19%, 37%, 31%, 11%, and 3%, for L1 through H3, respectively [28].

**Figure 4:**
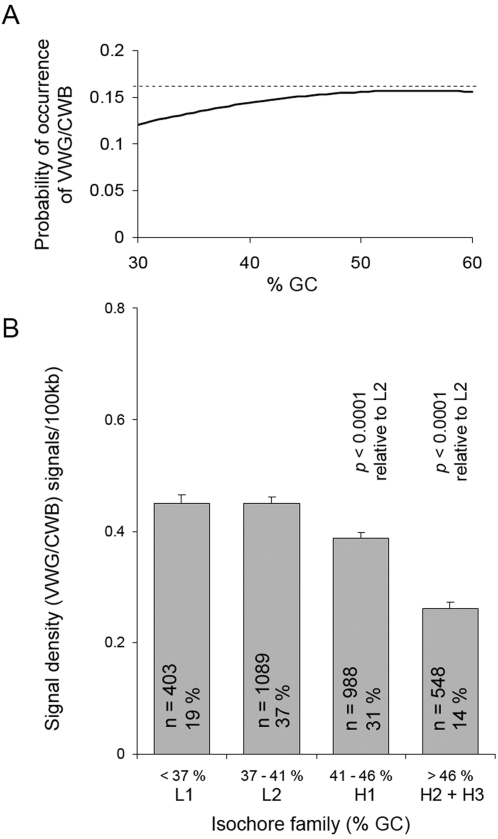
Dependence of VWG/CWB Occurrences and Signal Density on GC Content. (A) The probability of occurrence of the VWG/CWB motif with GC% over the physiological GC isochore range is shown. The horizontal dashed line at 0.16 serves as a reference straight line for comparison with the curve. (B) The VWG/CWB signal density in isochore families, L1, L2, H1, and H2+H3 is shown. The number of isochores analyzed (n), and the percentages of the genome that the isochore families contribute are given. Error bars indicate the standard error for each isochore family data set. The *P*-values from the two-tailed T-test listed above H1 and H2+H3 indicate that these isocore families/groups have mean signal densities that are significantly different from L2.

The probability of occurrence of the motif VWG/CWB at various %GC values is easily calculated if it is assumed that %A = %T and %G = %C, a reasonable approximation. These probabilities, over the GC isochore range from 30% to 60%, are shown in Fig. 4A. It can be seen that the probability of occurrence of the motif is lowest at the lowest GC% (30%). The probability of the motif occurrence gradually increases in value by about 30%, and then plateaus (up to 60% GC).

The probability of occurrence of the VWG/CWB motif in particular genomic DNA regions is expected to modulate the VWG/CWB signal density (number of signals per 100 kb), but a signal requires more than just the occurrence of the motif. A signal requires that VWG/CWB occurs with a period of ten nucleotides, and furthermore that highly regular dinucleosome length oscillations occur in the period-10 VWG/CWB count over 100 kb. In Fig. 4B we compute the signal density for the %GC ranges corresponding to the isochore families (see Table S4 for details). Interestingly, the signal density is nearly the same for L1 and L2, despite the increase in motif occurrence over this GC% range (Fig. 4A). It then decreases significantly at the high %GC values, despite the slight increase in the motif occurrence over this GC% range (Fig. 4A).

To examine how different GC contents affect the overall enrichment of non-transcribed sequences in VWG signal-rich 300 kb sequences over signal-poor 300 kb sequences, we computed the extents of enrichment for different isochore family combinations (Table 3). Chromosome Y was omitted from this analysis for the reasons stated below (Materials and Methods), and chromosome X was omitted because of its apparently anomalous signal content (Table 1). For all of the isochore families combined (100% of the genome, omitting chromosomes X and Y), we found a 2.4-fold enrichment, as described above. When isochore family L1 was omitted from the analysis, leaving the remaining isochore families L2-H3 (81% of the genome, omitting chromosomes X and Y), the enrichment increased to more than 5-fold. A nearly 5-fold enrichment also occurs for the 68% of the genome (omitting chromosomes X and Y) containing only the two most abundant isochore families, L2 and H1. Lastly, when only the high GC isochore families (H1-H3) were used (45% of the genome, omitting chromosomes X and Y), an 11-fold enrichment was obtained. Thus, the enrichment of non-transcribed 300 kb sequences in the signal-rich DNA persists for various combinations of GC isochore families. This result suggests that the enrichment found is not an indirect consequence of having correlations between non-transcribed 300 kb sequences and GC% combined with having similar correlations between signal density and GC%. Rather, there appears to be a direct correlation between high signal density and non-transcribed regions.

**Table 3:** Analysis of 300 kb sequences from the centers of signal-rich and signal-poor human genomic DNA regions for the absence of Ensembl-annotated transcripts and ESTs for different GC isochore family combinations.

Isochore families included (fraction of the genome)	No. of regions	Regions with no transcripts	*P*-value	Fold-enrichment (% signal-rich / -poor)
L1, L2, H1, H2, and H3 (100 %)				
Signal-rich	193	24 (12.4 %)	0.02	2.4
Signal-poor	275	14 (5.1 %)	0.04	
Total	468	38 (8.1 %)		
L2, H1, H2, and H3 (81 %)				
Signal-rich	149	14 (9.4 %)	0.01	5.3
Signal-poor	225	4 (1.8 %)	0.02	
Total	374	18 (4.8 %)		
L2 and H1 (68 %)				
Signal-rich	137	12 (8.8 %)	0.04	4.9
Signal-poor	166	3 (1.8 %)	0.01	
Total	303	15 (5.0 %)		
H1, H2, and H3 (45 %)				
Signal-rich	63	5 (7.9 %)	0.04	11.2
Signal-poor	141	1 (0.7 %)	0.01	
Total	204	6 (2.9 %)		

Chromosomes X and Y were excluded from the analysis. The criteria for signal-rich and signal-poor regions are described in the text. The *P*-values were calculated using the cumulative binomial distribution assuming the null hypothesis that there is no difference in the fractions of sequences that lack transcripts and ESTs between the signal-rich and signal-poor datasets. Isochore families: L1, <37 % GC; L2, 37–41 % GC; H1, 41–46 % GC; H2, 46–53 % GC; H3, >53 %, and the fraction of the genome for each isochore family were as described by Costantini et al. [28].

We similarly analyzed our correlation between low meiotic recombination and high VWG signal density using the same combinations of GC isochore families used above. We found that the number of signals per window for the RCSs on chromosomes 1–22 remained constant at about 0.29 for the first three GC isochore family combinations, significantly above the autosome average value of 0.21. For combined isochore families H1, H2, and H3, the number of signals per window for the RCSs increased to 0.31. The *P*-values for the differences found between the RCS value and the genome average value were <10^−27^ in all cases. Thus, the correlation that we found does not appear to arise indirectly as a consequence of having one correlation between ≥300 kb DNA VWG signal-rich regions and sequences with particular GC% values combined with another similar correlation between ≥300 kb DNA regions of low recombination activity and sequences with particular GC% values.

## Discussion

By analyzing the Ensembl annotations from one data set consisting of nearly continuous regions of size ≥300 kb having VWG chromatin organizing signals and another data set lacking these signals as a control, we found that the signal-containing data set was significantly enriched relative to the signal-lacking data set in 300 kb regions that lacked transcripts. This result suggests that 300 kb genomic DNA regions with no potential for transcription tended to acquire or maintain DNA signals to ensure the formation of an inactive chromatin structure. We also showed that large genomic DNA regions with low average recombination frequencies are enriched in VWG chromatin organizing signals. This finding supports the hypothesis proposed by Myers et al. [18] that recombination rates are constrained over large scales by physical stresses acting on the chromatin and/or by access of the DNA to the recombination machinery.

**Figure 5:**
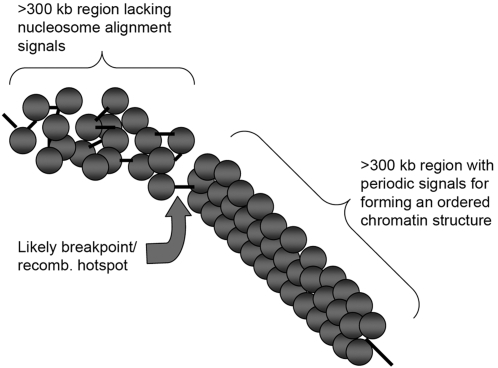
Chromatin Structure Model. The grey circles denote nucleosomes; the black lines denote linker DNA. The drawing illustrates the juxtaposition of a >300 kb well-ordered region of chromatin (by virtue of signals encoded in DNA) and a >300 kb region that is not highly ordered (because the DNA lacks signals). A likely breakpoint upon mechanical stress is indicated.

A chromatin structure model illustrating these ideas is shown in Fig. 5, which schematically depicts a large (>600 kb) region of chromatin where a sequence-defined ordered region of chromatin meets a region that lacks chromatin-organizing signals in the DNA. In this model, large (approximately >300 kb) DNA regions that are enriched in chromatin organizing signals with any physiological dinucleosome periodicity form more ordered chromatin structures than those regions that lack signals. A variety of different ordered chromatin structures is expected. Because the less ordered chromatin contains more configurational degrees of freedom than the ordered region, random physical stresses should tend to cause chromosome breakage in the less ordered chromatin. The large ordered region would tend to move slowly as a unit, and breakage would most likely occur near the junction between the two types of structures. Additionally, the DNA in the sequence-defined ordered chromatin structure should be less accessible to the recombination or transcription machinery.

Inspection of the chromosome-by-chromosome results for the relative abundances of VWG signals in large regions with low recombination frequency (RCSs) and in all sequences throughout the autosomes, suggests that chromosomes 5, 18, 21, and X may be exceptions to the general rule that RCSs are enriched in signals relative to other chromosome regions. Both chromosomes 18 and 21 have unusually high numbers of signals/window throughout each chromosome. These values are 0.24 and 0.25 for chromosomes 18 and 21, respectively, compared to the autosome average value of 0.21±0.02 signals/window. These high overall signal levels resulted in reductions in the signals/window values in RCSs due to the correction that we applied to account for local fluctuations in flanking region signal levels, as described above. If this correction is not applied, the signals/windows value for the combined chromosome 18 RCSs does exceed the autosome average value, and the *P-*value is less than 0.05. Small chromosome 21 contained only three RCSs, which provided only fifteen 100 kb windows for analysis. Therefore, the small sample size could be responsible for the apparent anti-correlation found in this case. Chromosome X has very low signal content value of 0.145 signals/window compared to the autosome average value of 0.21±0.02 signals/window. Conceivably, this difference between X and the autosomes reflects the unusual sequence content of X, which may facilitate the propagation of the inactivation signal in females [29], [30]. Lastly, chromosome 5 is unusual in that it contains many large abrupt changes in the VWG signal count over relatively small regions. For example, it contains six regions for which the VWG signal count per megabase increases or decreases by ≥9 counts over intervals ≤5 megabases, and three of these occur in the approximately 40 Mb short arm. In contrast, neither of the large chromosomes 1 or 2 contains any similarly abrupt VWG signal fluctuations of this magnitude. Interestingly, the short arm of chromosome 5 is associated with multiple breakpoint deletions, which result in cri-du-chat syndrome [31], consistent with breakpoints occurring near the junctions between ordered and disordered chromatin structures (Fig. 5).

Consistent with the low recombination frequencies within centromeres [19], [25], we found that alpha satellite DNA has two strong VWG chromatin signals. The 340 bp (dinucleosome) period value of one large Amplitude peak in Fig. 3 predicts a 170 bp nucleosome repeat, whereas the 396 bp peak predicts a 198 bp nucleosome repeat. The unusually short 170 bp nucleosome repeat value has been observed in centromeric chromatin [32], as well as a much longer approximately 198 bp nucleosome repeat [33]. Additionally it was experimentally determined that approximately half of the nucleosomes of alpha satellite chromatin are precisely phased with respect to the satellite repeat [34]. Thus, Fig. 3 is quite consistent with the experimental studies of centromeric chromatin. Our result suggests that alpha satellite DNA contains information for the formation of a distinctive chromatin structure in a portion of the alpha satellite chromatin, which may contribute to centromere function [35].

The persistence of our two correlations for various combinations of GC isochore families suggests that they are not merely secondary consequences of having two separate direct correlations. For example, there could be one correlation between the parameter involved and the GC content and another correlation between VWG signals and the GC content. In fact, the VWG signal density does not follow the changes with GC% expected simply from the increased occurrence of this motif at higher GC% values (Fig. 4 A). Rather, the VWG signal density significantly decreases at high GC% (Fig. 4 B). High GC% isochore families are gene rich [21]. Thus, gene-rich DNA may have fewer VWG chromatin signals than non-transcribed DNA, consistent with our result that 300 kb non-transcribed regions are enriched in VWG chromatin signals. It is interesting that omitting the AT-rich isochore family L1 improves the fold-enrichment of non-transcribed 300 kb regions in the signal-rich over the signal-poor datasets (Table 3). This result suggests that the VWG signal might function most effectively in regions of the genome having higher GC% values. Possibly, AT-rich motifs in DNA that can affect nucleosome formation such as AA/TT/TA [2] are used in AT-rich DNA.

Our finding that the frequency of occurrence of the period-10 VWG motif set (of 10 tri-nucleotides including the complements) is not unusually high for chromosome 1, and presumably for the whole genome, as was originally supposed [16] is somewhat surprising. However, VWG still appears to be special. The DNA structures, relative bendabilities, and nucleosome-forming potentials of DNA fragments containing various numbers and types of the VWG motifs periodically disposed at 10 bp intervals are not currently known. Nevertheless, the third column of Table 2 (the fraction of 100 kb windows that contain signals) suggests that part of the reason for why the VWG motif set is influential is that it leads to a dinucleosome period signal frequency that is not too high or too low. Clearly, a “signal” that is present nearly everywhere is not useful. Similarly, a “signal” that is rarely present is not very useful. Thus, a prerequisite for an influential signal may be that it must have an intermediate level of abundance (being present in, perhaps, approximately 0.20 to 0.50 of the 100 kb windows on the chromosome) so that it can be discriminating. The VWG motif set fulfills this requirement with a value of 0.20. In contrast, 6 of the 12 other motif sets studied were present in ≤0.13 of the 100 kb windows, while the other 6 were present in >0.50 of the 100 kb windows. For the small sampling that we did out of the very large number of possible triplet motif/complement motif sets consisting of 10–12 motifs each, only VWG gave a signal frequency in the intermediate range (between 0.20 and 0.50 of the 100 kb windows analyzed). This result suggests that, while there may be other influential motif sets besides VWG, only a relatively small percentage of possible motif/complement sets, containing 10 to 12 tri-nucleotides each, is likely contain signals that correlate with chromosome function.

Our findings suggest that there is information present in genomic DNA (long-range oscillations in period-10 VWG/CWB) that influences the formation of large-scale chromatin structures. We think that it is likely that the VWG signal is a part of the information encoded in genomic DNA that works by influencing the chromatin structures involved a variety of chromosome functions. For example, in addition to the mechanism illustrated in Fig. 5, discussed above, the unique arrangement of signal clusters along each chromosome, as in Fig. 1 C (black bars in lower diagram), could possibly serve as a “bar code” for the meiotic pairing of homologues. Aligned like-regions could make more Van der Waals chromatin fiber-chromatin fiber contacts between homologues, and could therefore lead to significantly stronger associations than those between unlike-regions. This association mechanism would not require nucleosomal DNA melting and subsequent base pairing between the two homologues to occur. DNA melting in nucleosomes is problematic because base pairing is greatly stabilized by the histones [26]. DNA sequence information affecting chromatin structure could also facilitate a variety of epigenetic mechanisms [36]. Our work provides the first evidence that particular periodic sequence motifs dispersed throughout human genomic DNA contribute to chromosome function.

## Materials and Methods

### Nature of the computational method

A continuous curve of period-10 triplet motif or triplet complement (motif/complement) was computed, as mentioned above. Briefly, the motif or its complement was identified on one DNA strand. The number of period-10 occurrences in a window ±51 nucleotides from each motif/complement position was determined. The period was allowed to vary within the range 10.00 to 10.33 by counting as periodic only nucleotide numbers which satisfy the relation
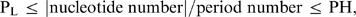
Where the nucleotide number is the integer number of nucleotides measured from any motif/complement position to another, the period number is the integer 1,2,3,4 or 5, P_L_ is the low end of the period variation and P_H_ is the high end. For example, the allowed (periodic) nucleotide numbers are: ±10; ±20; ±30, ±31; ±40, ±41; ±50, ±51. Thus, 41 is an allowed period number because 41/4 = 10.25, whereas 42 is not because 42/4 = 10.50.

The histogram data were then averaged in a sliding (5 nucleotide increments) 60 nucleotide window to generate a continuous curve of average period-10 motif/complement count versus nucleotide number. Closely similar results were obtained using window sizes over the range 50–150 nucleotides. A small correction using a sliding 600 nucleotide window was applied to take into account regions that were rich or poor in total motif/complement triplets as previously described [37], so that the variation in the period-10 motif/complement count over its local background value could be assessed. See reference 38 for more details. Recently, we showed that regions of DNA that have strong regular oscillations in VWG/CWB, as assessed by Fourier analysis, with period values in the dinucleosome length range (333 bp to 400 bp) generate highly ordered nucleosome arrays in chromatin assembled in vitro [38] and in mouse liver nuclei [17], [38], [39]. Thus, the signal in DNA can be regarded as being present every other nucleosome; this is sufficient to organize the nucleosome array. The signal strength was assessed from the Fourier transform of the oscillating curve of the motif/complement count versus the nucleotide number. Large Fourier amplitudes at a dinucleosome-length period value predict the nucleosome repeat value as the (dinucleosome-length period value)/2 [38], [39]. See reference 39 for a detailed explanation of what type of Fourier Amplitude peak constitutes a signal.

### Large-scale computational method

We collected non-overlapping 1-Mb GenBank files across each human chromosome (except for Y) from NCBI (Build 36) using the supercontigs provided. Chromosome Y was omitted from the analysis because it contained only 23 Mb of sequenced DNA in short contigs, and because the sequence is unusual, containing much repetitive DNA and large palendromes [40]. We wrote Perl programs to sequentially read in these files and compute from each a corresponding file consisting of the average VWG/CWB count at 5 nucleotide intervals. Each of these files was indexed to specify the chromosome number and the Mb file number. GenBank supercontigs less than 1 Mb and the remainders of supercontigs less than 1 Mb were inputted manually. From each of these files, 19 Fourier transforms (FTs) were computed using another Perl program and indexed for 100 kb windows that slid 50 kb per step, and were regarded as being statistically independent. For example, window #1 corresponded to nucleotide numbers 1 to 100000, #2 corresponded to nucleotide numbers 50000 to 150000, etc. Fourier amplitudes were computed every base pair throughout the physiological dinucleosome range, and 100 kb windows that met the criterion for having a strong VWG signal (see Results, Fig. 1A and B) were selected and maintained automatically by the program. Overlapping sequence files between two 1-Mb files were inputted manually when necessary. The data consisting of the presence or absence of a VWG signal at 50 kb increments was constructed for each chromosome using the supercontig lengths and gap lengths reported by NCBI.

### Other methods, data, and tools used in this study

Transcript annotations from 300 kb regions centered within all signal-rich or signal-poor regions from each chromosome (except Y) were obtained from Ensembl (http://www.ensembl.org). All types of transcripts annotated by Ensembl, as well as ESTs, were included in our analysis. In selecting the 300 kb regions that were contained within larger signal-rich and signal-lacking regions, either the central five100 kb windows that slid by 50 kb were chosen or, one window to the left of center was chosen when the larger region consisted of an even number of windows.

High resolution recombination rate data was provided by Myers et al. [18] (http://www.stats.ox.a .uk/mathgen/Recombination.html) where positions (SNP coordinates) and recombination rates (cM/Mb) are listed across each chromosome on average every 1000 bp. We wrote Perl programs to average the high resolution recombination frequency data [18], and to select 100 kb regions that had low recombination frequencies. Correction of the coordinates from the NCBI Build 34 used by Myers et al. to the current NCBI Build 36 was performed by using the UCSC Browser LiftOver tool (http://genome.ucsc.edu/cgi-bin/hgLiftOver).

GC Isochore coordinates and %GC values used in this study were from Costantini et al. [28]. These data were also used to calculate the GC% values of our signal-rich and signal-lacking 300 kb regions, and also of our recombination cold spots (RCSs). The fraction of the number of signals/window in each isochore throughout each human chromosome (except for Y), was also determined using our Supplementary Data Table S4 and the GC isochore data from Constantini et al. [28].

The degree of concordance between the VWG motif set and other triplet sets for chromosome 1 was determined in the following method. First, the presence (1) or absence (0) of a signal for each motif set was determined by computation for each 100 kb window sliding across the chromosome in 50 kb steps (the data for the VWG set alone is reported in Table S1). Second, for each window a pair-wise comparison was made, resulting in either signal concordance (0, 0 or 1, 1) or signal discordance (1, 0 or 0, 1), and the fraction of concordant windows was computed. Then, this fraction of concordant windows was compared with the fraction expected if there were no relation between the positions of the signals for the two sets of motifs (the null hypothesis). For example, for a motif set that resulted in 44% of the chromosome windows having a signal, compared to the VWG motif set with 21% of the windows having a signal, the null hypothesis expectation value fraction of concordant windows would be: (0.44)(0.21)+(1−0.44)(1−0.21) = 0.53. The significance of finding a fraction of concordant observations that differed from the null hypothesis expectation value was determined using the cumulative binomial distribution. *P*-values less than 0.05 indicate a significant degree concordance, *P*-values greater than 0.95 indicate a significant degree of discordance, and *P*-values between these limits indicate no significant relation between the chromosome positions at which signals occur for the two motif sets.

## Supporting Information

Table S1 Chromosome coordinates of the midpoints of the 100 kb windows that contain VWG signals across human chromosomes 1–22 and X.
(3.70 MB XLS)

Table S2 Chromosome coordinates of the central (or 25 kb left of center) 300 kb regions from the signal-rich (FT+), or signal-poor (FT-) data sets (see text for complete descriptions) across each human chromosome (except Y). The 300 kb regions lacking Ensembl-annotated transcripts and ESTs are indicated. The GC content of each 300 kb region is given.
(1.02 MB XLS)

Table S3 Analyasis of VWG signals in RCSs across each chromosome except for Y. The size, average cM/Mb, GC%, and average signal density of the flanking 1-Mb regions are given for each RCS.
(0.50 MB XLS)

Table S4 The number of VWG signals per 100 kb for each GC isochore for chromosomes 1–22 and X.
(0.82 MB XLS)

## References

[1] Collins FS, Green ED, Guttmacher AE, Guyer MS (2003). A vision for the future of genomics research.. Nature.

[2] Segal E, Fondufe-Mittendorf Y, Chen L, Thåström A, Field Y (2006). A genomic code for nucleosome positioning.. Nature.

[3] Peckham HE, Thurman RE, Fu Y, Stamatoyannopoulos JA, Nobel WS (2007). Nucleosome positioning signals in genomic DNA.. Genome Res.

[4] Pearson H (2006). Codes and Enigmas.. Nature.

[5] Finch JT, Klug A (1976). Solenoidal model for superstructure in chromatin.. Proc Natl Acad Sci U S A.

[6] Thoma F, Koller T, Klug A (1979). Involvement of histone H1 in the organization of the nucleosome and of the salt-dependent superstructure of chromatin.. J Cell Biol.

[7] Woodcock CL, Horowitz RA (1995). Chromatin organization re-viewed.. Trends Cell Biol.

[8] van Holde KE, Zlatanova J (1995). Chromatin higher order structure: chasing a mirage?. J Biol Chem.

[9] Dorigo B, Schalch T, Kulangara A, Duda S, Schroeder RR (2004). Nucleosome arrays reveal the two-start organization of the chromatin fiber.. Science.

[10] Schalch T, Duda S, Sargent DF, Richmond TJ (2005). X-ray structure of a tetranucleosome and its implications for the chromatin fibre.. Nature.

[11] Prunell A, Kornberg RD (1982). Variable center to center distance of nucleosomes in chromatin.. J Mol Biol.

[12] Woodcock CL, Grigoryev SA, Horowitz RA, Whitaker N (1993). A chromatin folding model that incorporates linker variability generates fibers resembling the native structures.. Proc Natl Acad Sci U S A.

[13] Zlatanova J, Leuba SH, Yang G, Bustamante C, van Holde K (1994). Linker DNA accessibility in chromatin fibers of different conformations: a reevaluation.. Proc Natl Acad Sci U S A.

[14] Stein A, Dalal Y, Fleury TJ (2002). Circle ligation of in vitro assembled chromatin indicates a highly flexible structure.. Nucleic Acids Res.

[15] Tsukiyama T (2002). The in vivo functions of ATP-dependent chromatin remodeling factors.. Nature Rev Mol Cell Biol.

[16] Baldi P, Brunak S, Chauvin Y, Krogh A (1996). Naturally occurring nucleosome positioning signals in human exons and introns.. J Mol Biol.

[17] Cioffi A, Fleury TJ, Stein A (2006). Aspects of large-scale chromatin structures in mouse liver nuclei can be predicted from the DNA sequence.. Nucleic Acids Res.

[18] Myers S, Bottolo L, Freeman C, McVean G, Donnelly P (2005). A fine-scale map of recombination rates and hotspots across the human genome.. Science.

[19] Choo KH (1998). Why is the centromere so cold?. Genome Res.

[20] Lynn A, Ashley T, Hassold T (2004). Variation in human meiotic recombination.. Annu Rev Genomics Hum Genet.

[21] Bernardi G, Bernardi G (2004). Structure and evolutionary genomics, Natural selection in genome evolution. New Comprehensive Biochemistry Volume 37,.

[22] Kong A, Gudbjartsson DF, Sainz J, Jonsdottir GM, Gudjonsson SA (2002). A high-resolution recombination map of the human genome.. Nature Genetics.

[23] Jensen-Seaman MI, Furey TS, Payseur BA, Lu Y, Roskin KM (2004). Comparative Recombination Rates in the Rat,Mouse, and Human Genomes.. Genome Res.

[24] Warburton PE, Waye JS, Willard HF (1993). Nonrandom localization of recombination events in human alpha satellite repeat unit variants: Implications for higher-order structural chracteristics within centromeric heterochromatin.. Mol Cell Biol.

[25] Laurent KH, Li M, Sherman S, Roizès G, Buard J (2003). Recombination across the centromere of disjoined and non-disjoined chromosome 21.. Human Mol Genet.

[26] van Holde KE (1989). Chromatin,.

[27] Int. Human Genome Seq. Consort (2001). Initial sequencing and analysis ot the human genome.. Nature.

[28] Costantini M, Clay O, Auletta F, Bernardi G (2006). An isochore map of human chromosomes.. Genome Res.

[29] Lyon MF (1998). X-chromosome inactivation: A repeat hypothesis.. Cytogenet Cell Genet.

[30] Wang Z, Willard HF, Mukherjee S, Furey TS (2006). Evidence of influence of genomic DNA sequence on human X chromosome inactivation.. PLoS Comput Biol.

[31] Medina M, Marinescu RC, Overhauser J, Kosik KS (2000). Hemizygosity of δ-catenin (CTNND2) is associated with severe mental retardation in cri-du-chat syndrome.. Genomics.

[32] Brown FL, Musich PR, Maio JJ (1979). The repetitive sequence structure of component α DNA and its relationship to the nucleosomes of the African green monkey.. J Mol Biol.

[33] Fittler F, Zachau HG (1979). Subunit structure of α-satellite DNA containing chromatin from African green monkey cells.. Nucleic Acids Res.

[34] Zhang X-Y, Fittler F, Horz W (1983). Eight different highly specific phases on α-satellite DNA in the African green monkey.. Nucleic Acids Res.

[35] Dalal Y, Furuyama T, Vermaak D, Henikoff S (2007). Inaugural Article: Structure, dynamics, and evolution of centromeric nucleosomes.. Proc Natl Acad Sci U S A.

[36] Goldberg AD, Allis CD, Bernstein E (2007). Epigenetics: A landscape takes shape.. Cell.

[37] Stein A, Bina M (1999). A signal encoded in vertebrate DNA that influences nucleosome positioning and alignment.. Nucleic Acids Res.

[38] Cioffi A, Dalal Y, Stein A (2004). DNA sequence alterations affect nucleosome array formation of the chicken ovalbumin gene.. Biochemistry.

[39] Dalal Y, Fleury TJ, Cioffi A, Stein A (2005). Long-range oscillation in a periodic DNA sequence motif may influence nucleosome array formation.. Nucleic Acids Res.

[40] Skaletsky H, Kuroda-Kawaguchi T, Minx PJ, Cordum HS, Hillier L (2003). The male-specific region of the human Ychromosome is a mosaic of discrete sequence classes.. Nature.

